# Spontaneous pneumothorax with coronavirus disease 2019 in non-ventilated patients: A single-center retrospective case series

**DOI:** 10.1016/j.amsu.2021.103134

**Published:** 2021-12-03

**Authors:** Jun Kawachi, Hiroshi Koyama, Yoshihisa Aida, Tadashi Kamio, Hiroshi Yamagami, Sho Nishiguchi

**Affiliations:** a Department of Surgery, Shonan Kamakura General Hospital, Japan; b Department of Critical Care, Shonan Kamakura General Hospital, Japan; c Department of General Internal Medicine, Shonan Kamakura General Hospital, Japan; d Department of Emergency, Shonan Kamakura General Hospital, Japan

**Keywords:** COVID-19, Intubation, Pneumothorax, Case series, SARS CoV-2

## Abstract

**Background:**

Pneumothorax is a rare complication of coronavirus disease 2019, and many of its associated factors are related to mechanical ventilation. We investigated the incidence and clinical features of patients with pneumothorax and coronavirus disease 2019 at a field hospital for patients who did not require intubation.

**Materials and methods:**

An isolated field hospital for COVID-19 patients who did not require ventilation was constructed. Patients who developed pneumothorax were extracted and reviewed retrospectively.

**Results:**

Between May 2020 and February 2021, 1061 patients were admitted to this field hospital. Among them, eight patients (0.75%, three men and four women) developed pneumothorax. The mean age at incidence was 79.9 (range: 20–96) years; all patients were over the age of 80 years, except one 20-year-old woman. Six of these eight patients (75%) died.

**Conclusion:**

Although pneumothorax is a rare complication of coronavirus disease-2019, it is predictive of a poor prognosis in older-adult patients.

## Highlights

• Incidence of pneumothorax in patients without mechanical ventilation was 0.75%.
• The mean age was 79.9 years and six of eight (75%) died.
• Pneumothorax is rare and predictive of poor prognosis patients with COVID-19.

## 1 Introduction

Since the end of 2019, severe acute respiratory distress syndrome coronavirus 2 (SARS-CoV-2) has spread worldwide. More than 1 billion people have been affected, and more than 2.5 million people have died [1].

Although a rare complication, some cases of pneumothorax in coronavirus disease-2019 (COVID-19) have been reported. Barotrauma is a well-known cause of pneumothorax in ventilated patients with diffuse alveolar injury due to pneumonia [2]. In February 2020, under the governance of the Kanagawa Prefecture, Japan, we started treating COVID-19 patients, and in May 2020, we developed a field hospital for COVID-19 patients who did not need intubation. This is the first report on the incidence and clinical features of patients with pneumothorax among non-ventilated patients with COVID-19.

## 2 Materials and methods

This was a single-center, retrospective, consecutive, and observational case series. An isolated field hospital for COVID-19 patients was constructed in the Kanagawa Prefecture next to Shonan Kamakura General Hospital, which managed this temporary hospital. The following were the target patients: patients with oxygen demand and patients without oxygen demand but who were elderly and with comorbidities that can be considered risks for severe COVID-19, including pulmonary disease, cardiovascular disease, chronic kidney disease, diabetes mellitus, and others [3,4]. Patients requiring intubation were transferred to other hospitals that specialized in the treatment of severely ill patients. Patients who did not wish to undergo intubation stayed at this hospital. All patients who were admitted to this field hospital were included in this study.

**Fig. 1:**
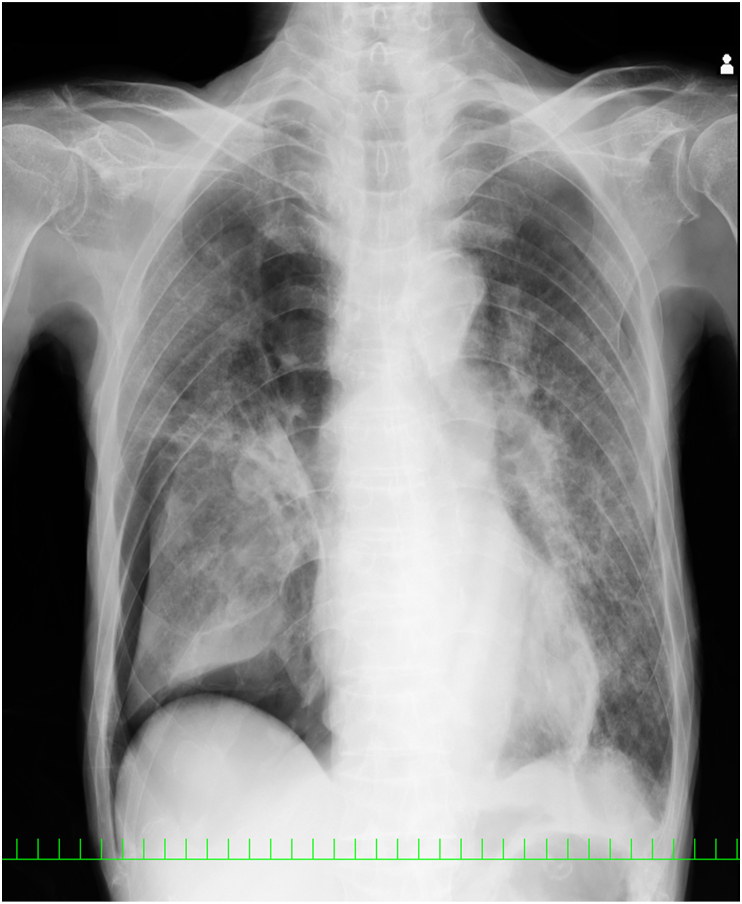
Chest radiograph of pneumothorax in coronavirus disease 2019 (patient 1).

**Fig. 2:**
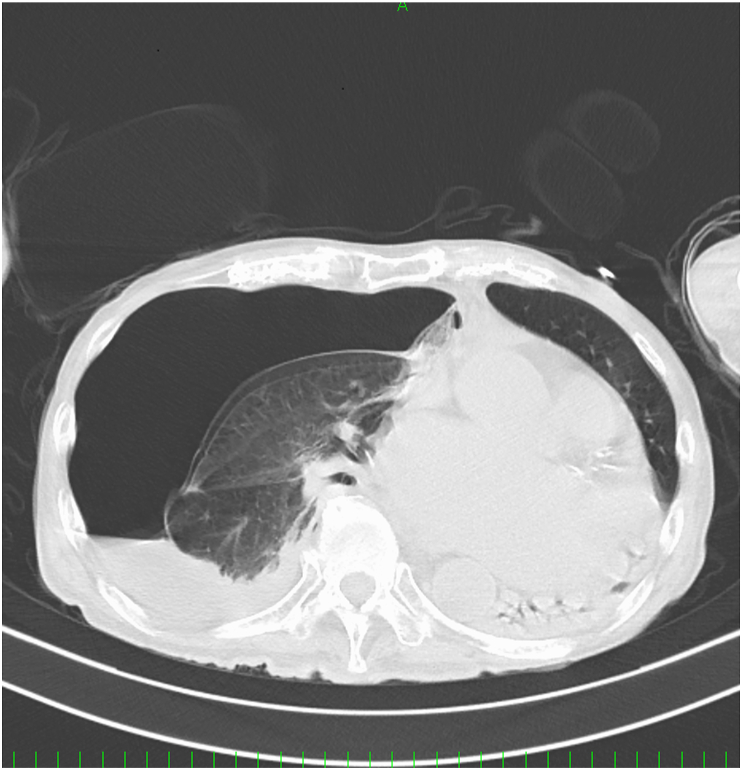
Computed tomography of pneumothorax in coronavirus disease 2019 (patient 7).

COVID-19 patients were diagnosed using polymerase chain reaction test or quantitative antigen test. All patients underwent computed tomography (CT) upon admission and on-demand by the physicians. The medications for pneumonia included dexamethasone, remdesivir, and favipiravir. We used nasal cannulas, oxygen masks, and high-flow nasal cannulas (HFNC) to treat patients; however, we did not use a ventilator or perform noninvasive positive-pressure ventilation (NPPV). Pneumothorax was diagnosed using chest radiography (Fig. 1) or CT (Fig. 2). The size of the pneumothorax was evaluated according to the guidelines of the American College of Chest Physicians: large is defined as >3 cm of collapse at the apex in chest radiography and small as <3 cm [5]. This case series has been reported in line with the PROCESS Guideline [6] and registered in Research Registry (www.researchregistry.com) (registration number: researchregistry7359).

## 3 Results

Of the 1061 patients admitted to the field hospital between May 18, 2020, and February 28, 2021, 599 (56.5%) were men and 462 (43.5%) were women. The patients had an average age of 68.4 (16–103) years. Eight patients had pneumothorax (three men and five women; Table 1), with an incidence rate of 0.75%. The mean age of patients with pneumothorax was 79.9 (20–96) years; all but one patient (age 20) were over 80 years old. All patients had preceding pneumonia on CT on admission, except for the 20-year-old patient (patient 8), who was considered to have a complicated primary spontaneous pneumothorax. Considering the laterality of pneumothorax, six were on the left side and two on the right. At the time of pneumothorax diagnosis, four patients were administered oxygen via nasal cannula, and the other four were not. Four patients required drainage with intercostal drain, and the other four were managed with conservative treatment.

**Table 1:** Clinical features of pneumothorax patients in coronavirus disease 2019.

No	Age	Sex	History	Smoking	Pneumonia	O2 (L/min)	side	size	ICD	Days from COVID-19 onset	Dex	Days from Dex initiation	Result	Days from pneumothorax until death
1	89	M	Parkinson HT	unknown	+	4	right	small	–	19	+	26	death	1
2	82	M	HT COPD	past	+	–	right	small	+	11	–	–	alive	–
3	88	F	HT TB dementia	unknown	+	–	right	small	–	4	+	3	death	3
4	96	F	HT COPD	unknown	+	–	right	small	–	37	+	32	death	15
5	88	F	Asthma CHF RA	none	+	0.5	right	large	+	20	+	21	death	3
6	94	F	Dementia tSAH	unknown	+	0.5	left	large	+	35	+	30	death	2
7	82	M	Prostate ca	past	+	1	right	large	+	22	+	7	death	32
8	20	F	none	none	–	–	left	small	–	14	–	–	alive	–

COVID-19, coronavirus disease 2019; HT, hypertension; COPD, chronic obstructive pulmonary disease; CHF, congestive heart failure; RA, rheumatoid arthritis; Dex, dexamethasone; tSAH, traumatic subarachnoid hemorrhage; ICD, intercostal drain; TB, tuberculosis.

Six of the eight patients (75%) died after an average of 9.3 (1–32) days from the onset of pneumothorax. The patients who died did not consent to intubation or resuscitation, although they received conservative treatment, including the administration of oxygen, remdesivir, favipiravir, antibiotics, anticoagulants, steroids, and nutrition therapy. The mean duration between the onset of COVID-19 and pneumothorax was 20.3 (4–37) days. Two (25%) patients were current smokers, and two (25%) were past smokers. The smoking status of the other four (50%) patients was unknown. Two (25%) patients had a history of chronic obstructive pulmonary disease. Six of the eight patients (75%) were administered dexamethasone, and the onset of pneumothorax occurred after an average of 19.8 (3–32) days from its initiation. Mortality in COVID-19 patients over 80 years of age with pneumothorax was high (85.7%, 6/7).

## 4 Discussion

In this study, we report that the incidence of pneumothorax among non-ventilated COVID-19 patients was 0.75% and the associated mortality was as high as 75%. Although the exact cause is unknown, spontaneous pneumothorax is believed to be caused by the rupture of the alveoli, which may be direct or through the mediastinum [7]. Secondary spontaneous pneumothorax can be due to weakness of the alveoli due to emphysema or necrotic pneumonia [8]. Moreover, acute respiratory distress syndrome or mechanical ventilation may also cause secondary pneumothorax [9]. Among COVID-19 patients, 1%–2% have pneumothorax [10,11]. In a report by Zantah [12], four of six patients with pneumothorax were under mechanical ventilation. Martinelli [13] reported that 38 of 60 patients (63.3%) with pneumothorax had invasive ventilatory support.

**Fig. 3:**
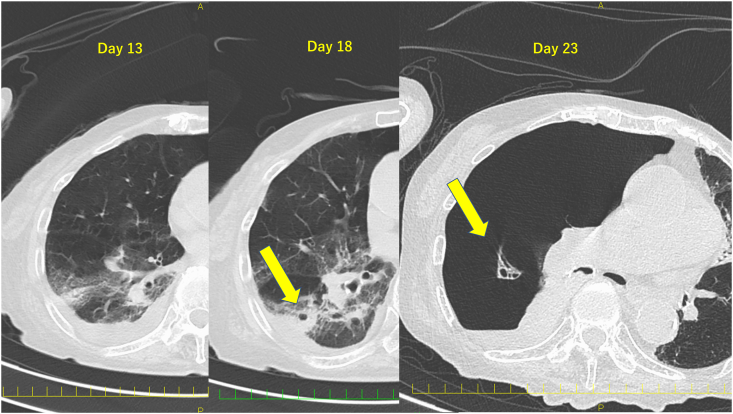
Cyst formation at the pneumothorax side of patient 5 (arrow).

**Fig. 4:**
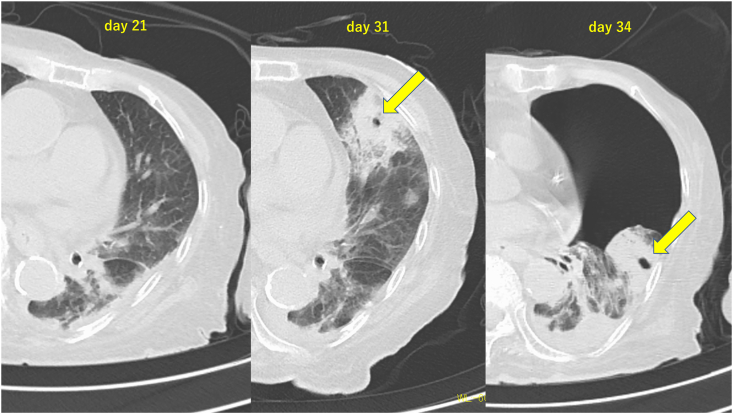
Cyst formation at the pneumothorax side of patient 6 (arrow).

**Fig. 5:**
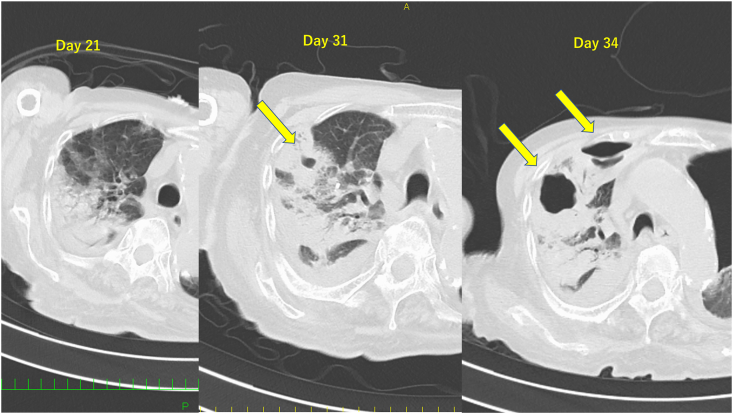
Cyst formation at the contralateral side of patient 6 (arrow).

At our institute, the incidence of pneumothorax was 0.75% (8/1061), and patients were not ventilated in principle owing to divided hospital function for moderately ill patients, as per the policy of the prefectural government. We did not conduct NPPV for infection control. None of the patients with pneumothorax used HFNC; therefore, we can exclude the influence of barotrauma. Alternatively, the main cause of the pneumothorax may be alveolar weakness since all patients, except one young woman, were over 80 years old and had high rates of steroid use (75%) and incidence of COPD (25%), smoking (50% current and past), and alveolar injury due to pneumonia. Everden [14] reported a case of pneumothorax and pneumomediastinum after cyst formation in a COVID-19 patient. In our study, cyst formation before the onset of pneumothorax was seen in two (patient 5 and 6) of three cases of large pneumothorax. Patient 5 was an 88-year-old female who had rheumatoid arthritis, chronic heart failure, and bronchial asthma. She suffered large pneumothorax in the right side. She died with respiratory failure three days later in spite of intercostal drainage. In the review, she got cyst formation before the onset of pneumothorax (Fig. 3). Patient 6 was a 94-year-old female who had dementia and a history of traumatic subarachnoid hemorrhage. She got large pneumothorax in left side which was treated with intercostal drain. She died from sepsis of unknown origin 30 days after the onset of pneumothorax. In the CT scan, cyst formation was identified before the onset of pneumothorax, not only in the affected side (Fig. 4) but also in the contralateral side (Fig. 5). These cysts may be the result of severe inflammation and may cause pneumothorax. The limitation of this study is the small number of patients with pneumothorax in a single institution, therefore we could not identify the risk factors for pneumothorax.

In acute Middle East respiratory syndrome, pneumothorax is associated with a poor prognosis [15]. Martinelli et al. [13] reported that the mortality rate of COVID-19 was significantly higher in patients over 70 years of age than in those below 70. At our institute, the overall mortality was 7.26% (77/1061), and that of patients over 80 years of age was 18.3% (61/334). The mortality rate of patients with pneumothorax over 80 years of age was 85.7% (6/7).

## 5 Conclusions

Although pneumothorax is a rare complication, it can be a predictive factor for poor prognosis in older adults with COVID-19. Further studies, including autopsy, are required to clarify the relationship between pneumothorax, COVID- 19, and comorbidities.

## Ethical approval

Approval of the research protocol: This retrospective study was approved by the ethics committee of Shonan Kamakura General Hospital.

## Sources of funding

This research did not receive any specific grant from funding agencies in the public, commercial, or not-for-profit sectors.

## Author contribution

All authors of this case report are involved in patient assessment, management, data collection, and the preparation of this article.

## Trial registry number

UIN: researchregistry7359

https://www.researchregistry.com/register-now#home/

## Guarantor

Jun Kawachi.

## Consent

Written informed consent was obtained from the patient for publication of this case report and accompanying images. A copy of the written consent is available for review by the Editor-in-Chief of this journal on request”.

## Data sharing and data accessibility

The data that support the findings of this study are available from the corresponding author upon reasonable request.

## Disclosure

Approval of the research protocol: This retrospective study was approved by the ethics committee of Shonan Kamakura General Hospital.

## Provenance and peer review

Not commissioned, externally peer-reviewed.

## Declaration of competing interest

There are no conflicts of interest to declare.
